# Association between smoking cessation and obstructive spirometry pattern among Korean adults aged 40–79 years

**DOI:** 10.1038/s41598-021-98156-9

**Published:** 2021-09-21

**Authors:** Yeo Jun Yoon, Myung Soo Lee, Kyu Won Jang, Jae Bum Ahn, Kyungduk Hurh, Eun-Cheol Park

**Affiliations:** 1 Premedical Course, Yonsei University College of Medicine, Seoul, Republic of Korea; 2 Department of Medicine, Yonsei University College of Medicine, Seoul, South Korea; 3 Department of Preventive Medicine, Yonsei University College of Medicine, 50-1 Yonsei-ro, Seodaemun-gu, Seoul, 03722 Republic of Korea; 4 Institute of Health Services Research, Yonsei University College of Medicine, 50-1 Yonsei-ro, Seodaemun-gu, Seoul, 03722 Republic of Korea

**Keywords:** Chronic obstructive pulmonary disease, Disease prevention, Public health

## Abstract

Smoking cessation aids in restoring lung function. However, whether long-term cessation can fully restore lung function has not been studied thoroughly, especially in Asian countries. This study aimed to evaluate the association between smoking cessation status and obstructive spirometry pattern among Koreans aged 40–79 years. In total, 6298 men and 8088 women aged 40–79 years from the Korea National Health and Nutrition Examination Survey (2015–2019) were analyzed for smoking cessation status, including the duration after quitting. Current-smokers showed a higher likelihood of having an obstructive spirometry pattern than never-smokers among both men (odds ratio [OR]: 3.15, 95% confidence interval [CI]: 2.32–4.29) and women (OR: 2.60, 95% CI: 1.59–4.23). In men, the effect tended to decrease with longer duration after cessation, but male ex-smokers who had quit smoking ≥ 20 years ago still showed a higher likelihood of having an obstructive spirometry pattern than male never-smokers (OR: 1.40, 95% CI: 1.05–1.89). In female ex-smokers, there was no significant association with the obstructive spirometry pattern, compared to that in female never-smokers. This study emphasizes the benefits of smoking cessation, possibility of long-lasting harm to lung function due to tobacco smoking, and importance of smoking prevention.

## Introduction

Chronic obstructive pulmonary lung disease (COPD) is a progressive life-threatening lung disease and a major public health problem worldwide^1^. Globally, 251 million cases of COPD are reported, and it accounts for approximately 5% of all deaths^2^.

The primary causative factor of COPD is tobacco smoking. Studies have revealed that 15–50% of elderly smokers eventually develop COPD, and 40–70% of COPD cases are attributed to tobacco smoking^3–6^.

Usually, lung function reaches its maximal capacity at the age of 20–25 years and thereafter declines gradually with aging^7,8^. Smoking accelerates this age-related decline in lung function and eventually results in chronic airway obstruction^8–10^. Particularly, greater smoking volume, or adolescent-onset smoking, is known to be associated with a high risk of lung function impairment and COPD^11–13^.

Smoking cessation could alleviate the accelerated decline in lung function, and prevent development and progression of COPD^12,14,15^. Three to five years after smoking cessation, the age-related FEV_1_ decline almost halved in ex-smokers compared to that in current smokers^13,16^. More recent studies have explored whether long-term smoking cessation could completely normalize lung function, and if so, how long it will take for full restoration. A meta-analysis reported that the FEV_1_ decline in ex-smokers did not differ from that in never-smokers^17^. In contrast, the lung function of ex-smokers did not normalize even decades after smoking cessation in a prospective cohort study conducted in the US population^18^. Moreover, it remains unclear whether the lung function of high-risk smokers, such as heavy smokers or adolescent-onset smokers, can recover to the level observed in other ex-smokers after long-term cessation. The impact of smoking cessation on lung function recovery has been well described in Western countries, while there are only few studies, conducted using reliable measuring instruments, focusing on long-term cessation in the Asian population.

Therefore, this study aimed to investigate the relationship between smoking cessation status, including the duration after cessation, and obstructive spirometry pattern among Korean adults aged 40–79 years, using a nationally representative survey. We also performed an additional analysis to investigate whether the cumulative smoking exposure or adolescent-onset smoking affected the association between lung function and smoking cessation status among male participants. Lastly, to minimize age differences across categories of smoking cessation status, separate analyses were performed after dividing the study participants into 10-year age groups.

## Results

Among 6298 men, 1111 (17.6%) were never-smokers; 2001 (31.8%) were current-smokers; and 1007 (16.0%), 493 (7.8%), 528 (8.4%), 477 (7.6%), and 681 (10.8%) were ex-smokers with ≥ 20, 15–20, 10–15, 5–10, and < 5 years of cessation, respectively. Among 8088 women, 7379 (91.2%) were never-smokers; 344 (4.3%) were current-smokers; and 123 (1.5%), 42 (0.5%), 74 (0.9%), 37 (0.5%), and 89 (1.1%) were ex-smokers with ≥ 20, 15–20, 10–15, 5–10, and < 5 years of cessation, respectively. Among male and female ex-smokers with ≥ 20 years of cessation, 787 (78.2%) and 120 (97.6%) had < 20 pack-years of smoking, respectively. On the other hand, 751 (37.5%) men and 281 (28.1%) women had < 20 pack-years of smoking among male and female current-smokers, respectively. Also, participants showed different age distributions across the smoking cessation status groups: Among men, the mean ages were 63.9 (SD, 9.7), 54.0 (SD, 9.6), and 57.9 (SD, 11.5) in ex-smokers with ≥ 20 of cessation, current-smokers and never-smokers, respectively; Among women, the mean ages were 53.3 (SD, 10.7), 53.2 (SD, 9.2), and 57.5 (SD, 10.4) in ex-smokers with ≥ 20 of cessation, current-smokers and never-smokers, respectively (Table 1).

**Table 1:** General characteristics of the study population.

Variables	Smoking cessation status																	
	Men									Women								
	Total	Never-smoker	Ex-smoker (years of cessation)					Current smoker	*P*-value	Total	Never-smoker	Ex-smoker (years of cessation)					Current smoker	*P*-value
			≥ 20	15–20	10–15	5–10	< 5					≥ 20	15–20	10–15	5–10	< 5		
	N (%)	N (%)	N (%)	N (%)	N (%)	N (%)	N (%)	N (%)		N (%)	N (%)	N (%)	N (%)	N (%)	N (%)	N (%)	N (%)	
Total	6298 (100.0)	1111 (17.6)	1007 (16.0)	493 (7.8)	528 (8.4)	477 (7.6)	681 (10.8)	2001 (31.8)		8088 (100.0)	7379 (91.2)	123 (1.5)	42 (0.5)	74 (0.9)	37 (0.5)	89 (1.1)	344 (4.3)	
**Cumulative smoking exposure (pack-years)**									< .0001									
< 20	3600 (57.2)	1111 (100.0)	787 (78.2)	281 (57.0)	252 (47.7)	183 (38.4)	235 (34.5)	751 (37.5)		7997 (98.9)	7379 (100.0)	120 (97.6)	42 (100.0)	68 (91.9)	33 (89.2)	74 (83.1)	281 (81.7)	
20–30	1141 (18.1)	N/A	124 (12.3)	98 (19.9)	106 (20.1)	97 (20.3)	170 (25.0)	546 (27.3)		54 (0.7)	N/A	1 (0.8)	0	3 (4.1)	4 (10.8)	6 (6.7)	40 (11.6)	
> 30	1557 (24.7)	N/A	96 (9.5)	114 (23.1)	170 (32.2)	197 (41.3)	276 (40.5)	704 (35.2)		37 (0.5)	N/A	2 (1.6)	0	3 (4.1)	0	9 (10.1)	23 (6.7)	
Age (years), mean ± SD	57.5 ± 10.7	57.9 ± 11.5	63.9 ± 9.7	59.2 ± 9.8	58.1 ± 10.0	57.2 ± 10.0	55.3 ± 10.7	54.0 ± 9.6	< .0001	57.3 ± 10.5	57.5 ± 10.4	53.3 ± 10.7	48.1 ± 8.7	51.8 ± 10.4	54.9 ± 10.8	53.9 ± 10.4	53.2 ± 9.2	< .0001
**School**									< .0001									< .0001
Middle school or below	1828 (29.0)	308 (27.7)	380 (37.7)	143 (29.0)	154 (29.2)	148 (31.0)	178 (26.1)	517 (25.8)		3306 (40.9)	3047 (41.3)	26 (21.1)	9 (21.4)	23 (31.1)	16 (43.2)	48 (53.9)	137 (39.8)	
High school	2091 (33.2)	301 (27.1)	290 (28.8)	163 (33.1)	167 (31.6)	151 (31.7)	229 (33.6)	790 (39.5)		2722 (33.7)	2425 (32.9)	51 (41.5)	21 (50.0)	28 (37.8)	13 (35.1)	29 (32.6)	155 (45.1)	
College or above	2379 (37.8)	502 (45.2)	337 (33.5)	187 (37.9)	207 (39.2)	178 (37.3)	274 (40.2)	694 (34.7)		2060 (25.5)	1907 (25.8)	46 (37.4)	12 (28.6)	23 (31.1)	8 (21.6)	12 (13.5)	52 (15.1)	
**Income**									0.0616									< .0001
Low	978 (15.5)	170 (15.3)	182 (18.1)	71 (14.4)	75 (14.2)	71 (14.9)	105 (15.4)	304 (15.2)		1619 (20.0)	1453 (19.7)	20 (16.3)	9 (21.4)	12 (16.2)	13 (35.1)	26 (29.2)	86 (25.0)	
Mid-low	1557 (24.7)	279 (25.1)	275 (27.3)	119 (24.1)	131 (24.8)	110 (23.1)	167 (24.5)	476 (23.8)		2024 (25.0)	1824 (24.7)	26 (21.1)	9 (21.4)	22 (29.7)	10 (27.0)	22 (24.7)	111 (32.3)	
Mid-high	1718 (27.3)	279 (25.1)	246 (24.4)	133 (27.0)	148 (28.0)	139 (29.1)	172 (25.3)	601 (30.0)		2103 (26.0)	1892 (25.6)	41 (33.3)	14 (33.3)	24 (32.4)	12 (32.4)	24 (27.0)	96 (27.9)	
High	2045 (32.5)	383 (34.5)	304 (30.2)	170 (34.5)	174 (33.0)	157 (32.9)	237 (34.8)	620 (31.0)		2342 (29.0)	2210 (29.9)	36 (29.3)	10 (23.8)	16 (21.6)	2 (5.4)	17 (19.1)	51 (14.8)	
**Occupation**^a^									< .0001									0.0157
White collar	1726 (27.4)	383 (34.5)	203 (20.2)	135 (27.4)	144 (27.3)	122 (25.6)	209 (30.7)	530 (26.5)		1340 (16.6)	1239 (16.8)	23 (18.7)	6 (14.3)	16 (21.6)	3 (8.1)	15 (16.9)	38 (11.0)	
Pink collar	574 (9.1)	87 (7.8)	72 (7.1)	32 (6.5)	40 (7.6)	55 (11.5)	59 (8.7)	229 (11.4)		1416 (17.5)	1259 (17.1)	25 (20.3)	10 (23.8)	15 (20.3)	6 (16.2)	18 (20.2)	83 (24.1)	
Blue collar	2535 (40.3)	399 (35.9)	390 (38.7)	208 (42.2)	219 (41.5)	190 (39.8)	253 (37.2)	876 (43.8)		1588 (19.6)	1476 (20.0)	16 (13.0)	7 (16.7)	12 (16.2)	4 (10.8)	11 (12.4)	62 (18.0)	
None/homemaker	1463 (23.2)	242 (21.8)	342 (34.0)	118 (23.9)	125 (23.7)	110 (23.1)	160 (23.5)	366 (18.3)		3744 (46.3)	3405 (46.1)	59 (48.0)	19 (45.2)	31 (41.9)	24 (64.9)	45 (50.6)	161 (46.8)	
**Residential area**									0.0716									0.0281
Capital	2601 (41.3)	437 (39.3)	419 (41.6)	217 (44.0)	215 (40.7)	193 (40.5)	289 (42.4)	831 (41.5)		3407 (42.1)	3070 (41.6)	65 (52.8)	24 (57.1)	40 (54.1)	18 (48.6)	44 (49.4)	146 (42.4)	
Metropolitan	1754 (27.9)	292 (26.3)	316 (31.4)	126 (25.6)	153 (29.0)	129 (27.0)	178 (26.1)	560 (28.0)		2246 (27.8)	2071 (28.1)	32 (26.0)	10 (23.8)	17 (23.0)	7 (18.9)	25 (28.1)	84 (24.4)	
Rural	1943 (30.9)	382 (34.4)	272 (27.0)	150 (30.4)	160 (30.3)	155 (32.5)	214 (31.4)	610 (30.5)		2435 (30.1)	2238 (30.3)	26 (21.1)	8 (19.0)	17 (23.0)	12 (32.4)	20 (22.5)	114 (33.1)	
**High-risk drinking**									< .0001									
No	5021 (79.7)	1006 (90.5)	888 (88.2)	407 (82.6)	417 (79.0)	377 (79.0)	527 (77.4)	1399 (69.9)		7775 (96.1)	7187 (97.4)	117 (95.1)	38 (90.5)	68 (91.9)	34 (91.9)	71 (79.8)	260 (75.6)	
Yes	1277 (20.3)	105 (9.5)	119 (11.8)	86 (17.4)	111 (21.0)	100 (21.0)	154 (22.6)	602 (30.1)		313 (3.9)	192 (2.6)	6 (4.9)	4 (9.5)	6 (8.1)	3 (8.1)	18 (20.2)	84 (24.4)	
**Physical activity**									0.0009									0.0070
Active	2849 (45.2)	545 (49.1)	468 (46.5)	237 (48.1)	247 (46.8)	219 (45.9)	309 (45.4)	824 (41.2)		3272 (40.5)	3021 (40.9)	46 (37.4)	22 (52.4)	31 (41.9)	15 (40.5)	28 (31.5)	109 (31.7)	
Inactive	3449 (54.8)	566 (50.9)	539 (53.5)	256 (51.9)	281 (53.2)	258 (54.1)	372 (54.6)	1177 (58.8)		4816 (59.5)	4358 (59.1)	77 (62.6)	20 (47.6)	43 (58.1)	22 (59.5)	61 (68.5)	235 (68.3)	
Height (cm), mean ± SD	169.2 ± 6.2	168.6 ± 6.3	167.6 ± 6.3	169.3 ± 5.8	168.9 ± 6.0	169.9 ± 5.7	170.3 ± 6.2	169.9 ± 6.3	< .0001	156.5 ± 5.9	156.3 ± 5.8	157.7 ± 5.7	159.3 ± 5.4	158.3 ± 6.7	156.2 ± 5.6	156.9 ± 5.8	158.3 ± 5.8	< .0001

*SD* standard deviation.

^a^Based on the International Standard Classification Occupations codes.

Current-smokers showed a higher likelihood of having an obstructive spirometry pattern than never-smokers among both men (odds ratio [OR]: 3.15, 95% confidence interval [CI]: 2.32–4.29) and women (OR: 2.60, 95% CI: 1.59–4.23). Male ex-smokers were more likely to have an obstructive spirometry pattern than male never-smokers; the risk tended to decrease with a longer duration of smoking cessation: the ORs were 1.93 (95% CI: 1.32–2.84), 2.66 (95% CI: 1.84–3.87), 1.48 (95% CI: 1.00–2.18), 1.18 (95% CI: 0.82–1.68), and 1.40 (95% CI: 1.05–1.89) in male ex-smokers with < 5, 5–10, 10–15, 15–20, and ≥ 20 years of cessation, respectively. Female ex-smokers showed no significant association with the obstructive spirometry pattern, when compared to female never-smokers. Other factors associated with obstructive spirometry pattern were greater pack-years of smoking (only in men), older age, residing metropolitan or rural area, physical inactivity (only in men), and higher height (Table 2).

**Table 2:** Association between smoking cessation status and obstructive spirometry pattern.

Variables	Obstructive spirometry pattern^a^			
	Men		Women	
	OR	95% CI	OR	95% CI
**Smoking cessation status (years of cessation)**				
Never-smoker	1.00		1.00	
Ex-smoker (≥ 20)	1.40	(1.05 1.89)	1.08	(0.44 2.67)
Ex-smoker (15–20)	1.18	(0.82 1.68)	1.79	(0.21 15.02)
Ex-smoker (10–15)	1.48	(1.00 2.18)	1.43	(0.48 4.24)
Ex-smoker (5–10)	2.66	(1.84 3.87)	1.62	(0.44 5.98)
Ex-smoker (< 5)	1.93	(1.32 2.84)	1.32	(0.47 3.69)
Current smoker	3.15	(2.32 4.29)	2.60	(1.59 4.23)
**Cumulative smoking exposure (pack-years)**				
< 20	1.00		1.00	
20–30	1.28	(1.02 1.62)	1.20	(0.42 3.43)
> 30	1.47	(1.20 1.80)	1.00	(0.35 2.85)
Age (years)	1.12	(1.10 1.13)	1.09	(1.08 1.11)
**Education level**				
Middle school or less	1.00		1.00	
High school	0.84	(0.69 1.02)	1.01	(0.77 1.34)
College or over	0.82	(0.64 1.05)	0.98	(0.66 1.45)
**Household income**				
Low	1.00		1.00	
Mid-low	1.08	(0.86 1.37)	0.92	(0.70 1.21)
Mid-high	0.90	(0.70 1.15)	0.83	(0.60 1.16)
High	0.91	(0.69 1.21)	0.76	(0.53 1.09)
**Occupation**^b^				
White collar	1.00		1.00	
Pink collar	1.12	(0.79 1.58)	1.41	(0.80 2.48)
Blue collar	1.12	(0.86 1.46)	1.33	(0.75 2.36)
None/homemaker	1.11	(0.85 1.45)	1.21	(0.71 2.04)
**Residential area**				
Capital area	1.00		1.00	
Metropolitan area	1.40	(1.15 1.70)	1.52	(1.13 2.05)
Rural	1.39	(1.14 1.69)	1.50	(1.13 1.99)
**High-risk drinking**				
No	1.00		1.00	
Yes	0.96	(0.78 1.18)	1.25	(0.68 2.30)
**Physical activity**				
Active	1.00		1.00	
Inactive	0.80	(0.69 0.92)	1.06	(0.85 1.32)
Height (cm)	1.04	(1.03 1.06)	1.05	(1.03 1.07)

*OR* odds ratio, *CI* confidence interval.

^a^Obstructive spirometry pattern was defined as an FEV_1_/FVC < 0.7.

^b^Based on the International Standard Classification Occupations codes.

Similarly, FEV_1_ and FEV_1_/FVC values showed an increasing tendency with longer duration of smoking cessation (Supplementary Table S1).

**Figure 1:**
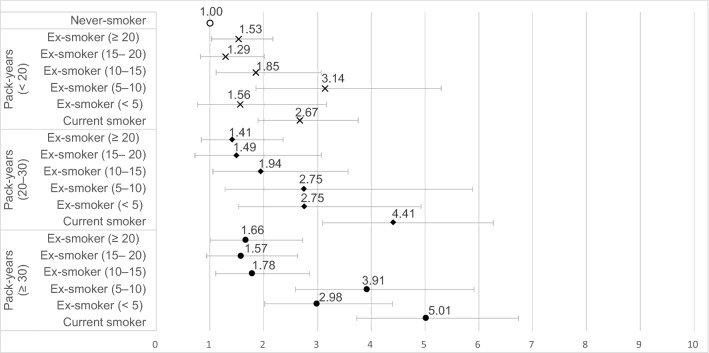
Association between smoking cessation status and obstructive spirometry pattern according to the cumulative smoking exposure in men. Adjusted for age, educational level, household income, occupation, residential area, physical activity, high-risk drinking, and height.

The ORs for the obstructive spirometry pattern were 2.67 (95% CI: 1.90–3.76), 4.41 (95% CI: 3.10–6.27), and 5.01 (95% CI: 3.73–6.74) in male current-smokers with < 20, 20–30, and ≥ 30 pack-years of smoking, respectively (comparison group: male never-smokers). Compared to male never-smokers, male ex-smokers with ≥ 20 years of cessation and < 20, 20–30, and ≥ 30 pack-years of smoking showed ORs of 1.53 (95% CI: 1.12–2.09), 1.41 (95% CI: 0.84–2.36), and 1.66 (95% CI: 1.01–2.72) for the obstructive spirometry pattern, respectively (Fig. 1).

**Figure 2:**
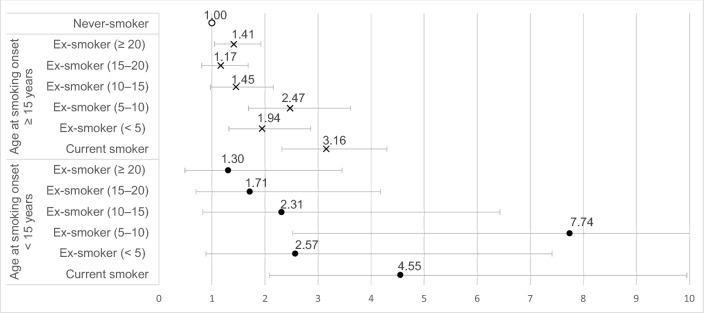
Association between smoking cessation status and obstructive spirometry pattern according to the age at smoking onset in men. Adjusted for cumulative smoking exposure, age, educational level, household income, occupation, residential area, physical activity, high-risk drinking, and height.

Compared to male never-smokers, male current-smokers who had started smoking before and after the age of 15 years showed ORs of 4.55 (95% CI: 2.08–9.94) and 3.16 (95% CI: 2.32–4.30), respectively, for the obstructive spirometry pattern. Male ex-smokers who had started smoking before and after the age of 15 years and had quit smoking ≥ 20 years ago showed ORs of 1.30 (95% CI: 0.49–3.45) and 1.41 (95% CI: 1.05–1.90), respectively, for the obstructive spirometry pattern (comparison group: male never-smokers) (Fig. 2).

Among men, the relationship between smoking cessation status and obstructive spirometry pattern was generally similar across age groups, except in the 40–49 years age group. Men aged 40–49 years did not show any significant association between smoking cessation status and obstructive spirometry pattern (Table 3).

**Table 3:** Association between smoking cessation status and obstructive spirometry pattern according to age group^a^.

Variables	Obstructive spirometry pattern^b^												
	Never-smoker	Ex-smoker (cessation years)										Current smoker	
		≥ 20		15–20		10–15		5–10		< 5			
	OR	OR	95% CI	OR	95% CI	OR	95% CI	OR	95% CI	OR	95% CI	OR	95% CI
**Age (years)**													
Men													
40–49	1.00	0.74	(0.18 3.01)	0.50	(0.11 2.32)	0.34	(0.08 1.40)	1.17	(0.42 3.26)	0.84	(0.32 2.22)	0.98	(0.45 2.12)
50–59	1.00	1.75	(0.84 3.64)	1.08	(0.47 2.47)	1.69	(0.75 3.81)	2.16	(0.93 5.02)	2.02	(0.95 4.31)	3.29	(1.76 6.17)
60–69	1.00	1.46	(0.92 2.33)	1.70	(1.02 2.83)	1.70	(0.94 3.05)	3.35	(1.89 5.93)	1.67	(0.91 3.05)	4.21	(2.62 6.77)
70–79	1.00	1.64	(1.03 2.60)	1.15	(0.60 2.19)	1.83	(0.93 3.62)	4.34	(2.03 9.30)	3.37	(1.66 6.83)	4.07	(2.19 7.56)
Women													
40–49	1.00	3.12	(0.75 12.91)	4.73	(0.86 25.99)	3.33	(0.63 17.74)	–	–	–	–	2.13	(0.73 6.25)
50–59	1.00	0.30	(0.04 2.30)	–	–	1.69	(0.18 15.81)	–	–	5.30	(1.11 25.38)	1.23	(0.46 3.33)
60–69	1.00	–	–	–	–	0.85	(0.16 4.38)	–	–	0.98	(0.22 4.35)	3.78	(1.47 9.73)
70–79	1.00	1.08	(0.25 4.73)	–	–	–	–	6.90	(1.15 41.45)	0.89	(0.11 7.46)	5.11	(1.52 17.21)

*OR* odds ratio, *CI* confidence interval.

^a^Adjusted for all covariates (cumulative smoking exposure, age, educational level, household income, occupation, residential area, physical activity, high-risk drinking, and height).

^b^Obstructive spirometry pattern was defined as an FEV_1_/FVC < 0.7.

## Discussion

In this cross-sectional study, we evaluated the association between smoking cessation status, considering the duration after smoking cessation, and obstructive spirometry pattern among Korean adults aged 40–79 years. Our findings suggested that a longer duration of smoking cessation was related to a decreasing tendency in the likelihood of having an obstructive spirometry pattern as well as an improvement of lung function parameters, among men. However, despite ≥ 20 years after smoking cessation, male ex-smokers still showed a higher likelihood of having the obstructive spirometry pattern than male never-smokers. Analyses after stratification by age groups, which reduced the potential difference in age distribution across smoking cessation status categories, showed a tendency similar to that observed in the main analysis. In women, the number of ex-smokers with the obstructive spirometry pattern was not sufficient to perform analyses.

Since our study was cross-sectional design, the partial restoration of lung function observed can be explained in two ways. First, although the rate of lung function decline normalized, the decrease in maximal lung function due to smoking was not fully restored. Second, acceleration of the age-related decline in lung function due to smoking could not be fully normalized even after long-term cessation. Our results are consistent with recent cohort study that although smoking cessation has a benefit in terms of lung function, lung injury from smoking could persist for decades after smoking cessation^18^.

Tobacco smoking is related to pathophysiologic abnormalities of the lung, including inflammation^19–21^, immune dysfunction and increased susceptibility to infection^22–24^, mucus hypersecretion^25,26^, genetic abnormalities^27,28^, and airway remodeling^29–31^. Although such lung abnormalities can improve with smoking cessation, there is evidence for sustained pathophysiological abnormalities in ex-smokers^19,21^. Lung injuries during the active smoking period, such as irreversible emphysematous change, may also contribute to persistent lung function impairment.

Findings regarding heavy smokers or adolescent-onset smokers are also notable. In male current-smokers, greater cumulative smoking exposure and adolescent-onset smoking showed relatively higher effect sizes (represented as ORs) for the obstructive spirometry pattern than their counterparts. However, among male ex-smokers with a smoking cessation duration of ≥ 20 years, participants showed similar effect sizes for the obstructive spirometry pattern across categories of cumulative smoking exposure and age at smoking onset. Therefore, long-term smoking cessation may be more beneficial for high-risk smokers than for low-risk smokers, in terms of recovering lung function. These findings reinforce prior research that heavy smokers benefited from smoking cessation more than did light smokers, in the first year after cessation^13^.

This study has several limitations. First, the number of years of smoking cessation and cumulative smoking exposure were indirectly calculated without considering intermittent smoking history. Although self-reported smoking history is known to be highly reliable, the gap between the actual and estimated smoking history may have affected our results. Second, Korean women are likely to underreport their history of smoking owing to social unacceptance. Therefore, the number of female smokers was lower than expected in this study, and the results may have been distorted. Third, with the cross-sectional design, we could not estimate individuals’ age-related decline in lung function. Instead, the likelihood of having an obstructive spirometry pattern was used to evaluate the lung function of participants. The possibility of unmeasured confounding due to factors such as passive smoking, occupational exposure to harmful particles, or individual variation in metabolic enzyme activity should also be considered^32,33^. Thus, our results should be cautiously interpreted, when compared to the findings of similar studies. Further long-term prospective study, including accurate smoking history of women participants, is needed to evaluate impacts of smoking cessation on age-related decline in lung functions in Koreans.

Despite these limitations, this study has a major strength: this study was based on one of the most representative health statistics of the Korean population and a reliable measurement of lung function.

In conclusion, our study showed that a longer duration of smoking cessation was linked to a decreasing tendency in the likelihood of having an obstructive spirometry pattern among men. Our findings suggest that tobacco smoking causes long-lasting harm to lung function and indicates the importance of the prevention and cessation of smoking, particularly in high-risk male smokers.

## Methods

### Ethical considerations

The study data were collected from the Korea National Health and Nutrition Examination Survey (KNHANES), which is conducted by the Korean government. The KNHANES data are anonymized and publicly available for research. Thus, this study was approved as an exemption by the Institutional Review Board of Yonsei University’s Health System (IRB No: 4-2021-0663).

### Study subjects and data sources

This was a cross-sectional study. The study data were collected during 2015–2019 from the KNHANES VI, VII, and VIII. The KNHANES is a cross-sectional, nationally representative survey that assesses the health, risk factors for health, and nutritional status of Koreans; it is conducted annually by the Korea Centers for Disease Control and Prevention (KCDC). Details on the design and contents of the KNHANES are available on the KNHANES webpage (https://knhanes.kdca.go.kr/knhanes/eng/index.do).

The study included participants aged 40–79 years, which is the target population of the spirometry test in the KNHANES. Participants with asthma, with unreadable spirometry results, or who were unable to undergo the test were excluded from the study. Those who had incomplete or missing data were also excluded. The final study population included 6298 men and 8088 women.

### Measures

The outcome variable was obstructive spirometry pattern, defined as an FEV_1_/FVC < 0.7^34,35^. Using pre-bronchodilator spirometry, lung function was measured at least two times for each participant and the largest value was reported.

Spirometry was performed by trained technicians, using the American Thoracic Society (ATS) /European Respiratory Society (ERS) 2005 standards^36,37^. The study included only valid spirometry results that met ATS/ERS acceptability and repeatability criteria: (1) two or more spirometry curves should be free from artefacts, have good starts and show at least 6 s of exhalation; (2) two largest values of FEV_1_ or FVC should be within 150 mL of each other^36^. The quality of the spirometry test was managed by the Korean Academy of Tuberculosis and Respiratory Diseases^38^.

Participants were classified as never-smokers, ex-smokers, or current-smokers according to their self-reported smoking status. Ex-smokers were subdivided into five categories according to the duration after smoking cessation: < 5 years, 5–10 years, 10–15 years, 15–20 years, and ≥ 20 years.

The covariates were selected based on the previous literature. Pack-years of smoking (< 20 years, 20–30 years, > 30 years), age (continuous variable), height (continuous variable)^39^, high-risk drinking (yes, no)^40,41^, physical activity (active, inactive)^42^, and socioeconomic factors such as education level (middle school or below, high school, college or above), household income (quartile of household income according to the 2015–2019 KNHANES survey), occupation (white collar, pink collar, blue collar, none or homemaker), area of residence (capital, metropolitan, rural) were included as covariates^43,44^. High-risk drinking was defined as having more than seven (men) or five (women) drinks at one time, at least twice per week^45,46^. Physical activity was evaluated using Global Physical Activity Questionnaire (GPAQ), developed by World Health Organization^47,48^.

### Statistical analyses

All analyses were performed separately by gender, considering gender-specific differences in smoking rate and smoking effect on lung function^49,50^. We compared the prevalence of obstructive spirometry pattern in current-smokers, in ex-smokers with various duration of cessation to that in never-smokers.

Chi-square tests (for categorical variables) and one-way analysis of variance (for continuous variables) were performed to determine differences in general characteristics between participants according to their smoking cessation status. Multiple logistic regression analysis was performed to calculate ORs with 95% CIs for evaluating the relationship between smoking cessation status and obstructive spirometry pattern. Additionally, multiple linear regression analyses were conducted to identify whether smoking cessation status was related to FEV_1_, FVC, or FEV_1_/FVC.

The data analysis for this paper was generated using SAS software, Version 9.4 of the SAS System for Unix. Copyright © 2016 SAS Institute Inc. SAS and all other SAS Institute Inc. product or service names are registered trademarks or trademarks of SAS Institute Inc., Cary, NC, USA.

## Supplementary Information

Supplementary Table S1.

## Data Availability

The datasets generated during and/or analysed during the current study are available in the KNHANES webpage (Korean), [https://knhanes.kdca.go.kr/knhanes/sub03/sub03_02_05.do].
