# Supplementary material for: Association between smoking cessation and obstructive spirometry pattern among Korean adults aged 40–79 years
Source: Sci Rep. 2021 Sep 21;11:18667. doi: 10.1038/s41598-021-98156-9 (PMC8455662; doi:10.1038/s41598-021-98156-9)
Supplement: Supplementary file 1 — Supplementary Table S1. [file 41598_2021_98156_MOESM1_ESM.docx]

**Supplementary Materials**

| **Supplementary Table S1. Association between smoking cessation status and spirometry parameters** | | | | | | | | | | | | |
| --- | --- | --- | --- | --- | --- | --- | --- | --- | --- | --- | --- | --- |
| **Variables** | | **Spirometry parameters** | | | | | | | | | | |
|  |  | **FEV_1_^a^ (mL)** | | |  | **FVC^a^ (mL)** | | |  | **FEV_1_/FVC^a^ (%)** | | |
|  |  | **β** | **S.E.** | **p-value** |  | **β** | **S.E.** | **p-value** |  | **β** | **S.E.** | **p-value** |
| **Men** | **Smoking cessation status (years of cessation)** | | | | | | | | | | | |
|  | Never-smoker | Ref. |  |  |  | Ref. |  |  |  | Ref. |  |  |
|  | Ex-smoker (≥ 20) | -3.4 | 22.0 | 0.8778 |  | 21.9 | 25.0 | 0.3815 |  | -0.5 | 0.3 | 0.1093 |
|  | Ex-smoker (15–20) | 4.8 | 25.2 | 0.8481 |  | 29.7 | 30.2 | 0.3259 |  | -0.4 | 0.4 | 0.3065 |
|  | Ex-smoker (10–15) | 3.6 | 27.6 | 0.8963 |  | 72.9 | 32.4 | 0.0248 |  | -1.4 | 0.4 | 0.0003 |
|  | Ex-smoker (5–10) | -54.7 | 27.1 | 0.0447 |  | 31.1 | 33.2 | 0.3489 |  | -2.1 | 0.4 | <.0001 |
|  | Ex-smoker (< 5) | -86.5 | 25.2 | 0.0006 |  | -27.1 | 29.0 | 0.3512 |  | -1.9 | 0.4 | <.0001 |
|  | Current smoker | -78.2 | 21.4 | 0.0003 |  | 37.3 | 24.8 | 0.1338 |  | -2.8 | 0.3 | <.0001 |
| **Women** | **Smoking cessation status (years of cessation)** | | | | | | | | | | | |
|  | Never-smoker | Ref. |  |  |  | Ref. |  |  |  | Ref. |  |  |
|  | Ex-smoker (≥ 20) | -31.6 | 30.3 | 0.2976 |  | -35.3 | 35.1 | 0.3141 |  | -0.3 | 0.5 | 0.5869 |
|  | Ex-smoker (15–20) | 17.5 | 34.7 | 0.6144 |  | 27.9 | 41.8 | 0.5040 |  | -0.2 | 0.8 | 0.7717 |
|  | Ex-smoker (10–15) | -1.9 | 42.4 | 0.9644 |  | -6.4 | 46.7 | 0.8912 |  | -0.1 | 0.7 | 0.8950 |
|  | Ex-smoker (5–10) | -97.6 | 70.5 | 0.1667 |  | -82.5 | 75.4 | 0.2745 |  | -1.4 | 0.9 | 0.1164 |
|  | Ex-smoker (< 5) | -35.4 | 35.7 | 0.3225 |  | -11.6 | 41.4 | 0.7787 |  | -0.9 | 0.7 | 0.1942 |
|  | Current smoker | -0.1 | 22.0 | 0.9946 |  | 36.5 | 24.3 | 0.1344 |  | -1.1 | 0.4 | 0.0015 |
| ^a^Adjusted for cumulative smoking exposure, age, educational level, household income, occupation, residential area, physical activity, high-risk drinking, and height | | | | | | | | | | | | |
